# Safety of long-term creatine supplementation in women's football players: a real-world in-season study

**DOI:** 10.1080/15502783.2025.2591782

**Published:** 2025-12-02

**Authors:** Murilo Perez Garcia, Igor Longobardi, Tieme Saito, Matheus Santos Miranda, Hamilton Roschel, Bruno Gualano

**Affiliations:** a Applied Physiology and Nutrition Research Group – School of Physical Education and Sport and Faculdade de Medicina FMUSP, Universidade de Sao Paulo, Sao Paulo, SP, Brazil; b Center of Lifestyle Medicine, Faculdade de Medicina FMUSP, Universidade de Sao Paulo, São Paulo, SP, Brazil; c Sport Club Corinthians Paulista, São Paulo, Brazil; d Laboratory of Assessment and Conditioning in Rheumatology; Hospital das Clínicas HCFMUSP, Faculdade de Medicina FMUSP, Universidade de Sao Paulo, São Paulo, SP, Brazil; e Rheumatology Division, Hospital das Clinicas HCFMUSP, Faculdade de Medicina, Universidade de São Paulo, Sao Paulo, SP, Brazil

**Keywords:** Sports nutrition, soccer, kidney, liver, dietary supplements

## Abstract

Although creatine supplementation is well established for enhancing athletic performance, data on its long-term safety are still limited, particularly among female athletes. This study investigated the effects of in-season creatine supplementation on biochemical safety markers in young female football players. This real-world, longitudinal single-arm study assessed the safety of creatine supplementation during a competitive season in 71 female athletes from youth and professional football teams. Participants received 20 g/day of creatine monohydrate for 7 days, followed by 5 g/day for the remainder of the season. Dietary intake and a comprehensive panel of hematological, renal, and hepatic biomarkers were evaluated at baseline, mid- (week 16), and end-season (week 32). Linear mixed-model with repeated measures analysis revealed that 8 out of 18 biochemical markers showed statistically significant though clinically minor fluctuations throughout the season. All analytes, except creatine phosphokinase (CPK), remained within reference ranges. No adverse effects were observed on renal (e.g. glomerular filtration rate, creatinine, urea, albuminuria) or hepatic (ALT, AST) function. CPK levels variation likely reflected training load rather than supplementation effects. In this single-arm in-season cohort, long term creatine supplementation was not associated with clinically meaningful derangements in biochemical safety markers in female football players. These findings support the long-term safety profile of creatine in this population and encourage further research into its sex-specific effects in athletic settings.

## 1 Introduction

Creatine is a naturally occurring guanidine compound that plays a central role in cellular energy metabolism [1]. Even though the human body synthesizes creatine endogenously, it can also be obtained through diet (primarily animal-based foods such as red meat, fish, and poultry). Since the seminal work by Harris et al. [2], demonstrating that creatine supplementation increases intramuscular creatine levels, it has become one of the most extensively studied dietary supplements both at the clinical and athletic performance setting.

A growing body of literature supports the benefits of creatine across a wide range of clinical conditions and sports [3,4], including football [5]. Claudino et al. [6], in a randomized placebo-controlled trial, demonstrated that creatine supplementation (20 g/day for one week, followed by 5 g/day for six weeks) prevented declines in lower-limb muscle power in elite soccer players during the pre-season. Furthermore, Cox et al. [7] found that six days of creatine supplementation (20 g/day) improved repeated sprint ability and agility in elite female football players from the Australian National Team. Similarly, Ramírez-Campillo et al. [8] reported that amateur female football players undergoing plyometric training experienced greater improvements in jumping and repeated sprint performance with creatine supplementation (20 g/day for one week, followed by 5 g/day for five weeks) compared to placebo. Supporting these findings, a systematic review and meta-analysis concluded that creatine has a large and significant positive impact on anaerobic performance in football players [5], which is particularly relevant given the primary physical demands of the sport (e.g. sprinting, jumping, and changing direction) [9].

Although creatine supplementation is highly recommended for female athletes [10] due to its well-established ergogenic benefits, a lack of long-term, real-world studies evaluating its safety profile, particularly among female athletes still remains. Research conducted during the competitive season (i.e. in-season) is especially limited, largely due to the practical and logistical constraints of working with athletes during this demanding period. This is a critical gap, as sex-specific hormonal, metabolic, and physiological factors may influence both the efficacy and safety of creatine supplementation in this population [11,12]. For instance, serum concentrations of several analytes related to endocrine, immune, metabolic, and other biological processes have been linked to sex and female hormonal status [13]. Moreover, female sex hormones also influence fluid dynamics and electrolyte balance [14], with potential implications for cardiovascular and renal function [15]. In addition, different phases of the menstrual cycle have been associated with subtle variations in creatinine clearance [16]. Since creatine supplementation directly affects creatinine metabolism [17], it remains unclear whether such fluctuations stay within reference ranges in female athletes using creatine. Addressing these gaps is essential for informing evidence-based recommendations. Therefore, the aim of this study was to assess the safety of in-season creatine supplementation in female football players. We hypothesized that creatine supplementation would not adversely affect safety biomarkers in female athletes.

## 2 Methods

Procedures were approved by the local Ethical Review Board (CAAE: 74940423.2.0000.0068) and complied with the standards established by the 1964 Helsinki Declaration. Informed consent (or assent) was obtained from all participants and, when applicable, from their legal guardians.

### 2.1 Participants

Seventy-one female football players from the under-17 (U17; *n* = 13), under-20 (U20; *n* = 25), and professional (PRO; *n* = 33) categories of the same football team from São Paulo, Brazil (Sport Club Corinthians Paulista) participated in this study. Participants aged ≥ 16 years and without any medical condition known to affect outcomes of interest (e.g. pregnancy, kidney or hepatic diseases) were considered eligible. Athletes unavailable to comply with study protocol or with a recent history of creatine supplementation (<30 days) were excluded from the study.

### 2.2 Study design

This was an exploratory quasi-experimental study employing a single-arm, pre-to-post design, carried out in-season across various age categories of the Brazilian Women’s Football Championship. Dietary assessments and biochemical exams were performed immediately before (T0; baseline) the beginning of the season and reassessed after 16 (T1; mid-season) and 32 weeks (T2; end-season) of dietary supplementation.

### 2.3 Procedures

#### 2.3.1 Creatine supplementation

In line with established supplementation protocols [18], creatine was administered using a two-phase strategy: a loading phase of 20 g/day of creatine monohydrate (Creavitalis, 99.9% purity; Alzchem Trostberg GmbH, Germany) for the first 7 days, followed by a maintenance phase of 5 g/day for the remainder of the study. Dietary supplements were provided in unlabeled, indistinguishable packages containing 5-g tablets of similar shape, taste, color, and odor. Participants were advised to consume four 5-g doses divided into their main meals (i.e. breakfast, lunch, an afternoon snack, and dinner) during the first week, and then a single 5-g dose once a day until the end of the season. Adherence to supplementation was closely monitored through daily verification by the club’s dietitian.

#### 2.3.2 Laboratory analysis

Biochemical analyzes were performed in blood and urine samples, collected by a registered nurse, in the morning following a 10-h overnight fast. Hematological parameters were assessed using automated methods based on electrical impedance, calorimetry, and fluorescence, with microscopic evaluation when required. Aspartate aminotransferase (AST) and alanine aminotransferase (ALT) were quantified by UV spectrophotometry. Serum ferritin was measured by chemiluminescence. Creatine phosphokinase (CPK) activity was measured by an optimized kinetic method. Serum creatinine was determined by the Jaffé method without deproteinization. Urea was measured enzymatically using the urease-glutamate dehydrogenase method with UV detection. Sodium (Na^+^) and potassium (K^+^) were assessed by indirect ion-selective electrodes. Urinary albumin (i.e. albuminuria) was determined by immunoturbidimetry from first-morning samples as recommended [19]. All analyzes were performed by an external referral laboratory (Delboni Medicina Diagnóstica) accredited by Brazilian Society of Clinical Pathology/Laboratory Medicine (control number: 64012951).

Estimated glomerular filtration rate (eGFR) was calculated using the 2021 CKD-EPI creatinine equation adjusted for body surface area [20].

#### 2.3.3 General assessments

Anthropometrics were performed by an accredited evaluator blinded to the study protocol. Body mass was measured using a calibrated digital scale with an accuracy of 0.1 kg (AVA−350; Avanutri, Sao Paulo, Brazil); measurements were taken before any physical activity, with participants barefoot and wearing the club’s training uniform for standardization.

Menstrual cycle phase was monitored using a self-reported online questionnaire, which recorded the symptomatology, as well as the onset and duration of the periods of menstruation and non-menstruation.

#### 2.3.4 Dietary intake

Due to scheduling inconsistencies during the regular season, nutritional data could not be assessed in PRO players. U17 and U20 participants received both oral and written instructions from a registered dietitian on how to complete 3-day food records, consisting of two non-consecutive weekdays and one weekend day (all regular non-match nor prior-match days). The dietitian also provided guidance on estimating portion sizes using household measures [21]. Dietary intake, including total energy (TEI) from macronutrients [protein (PRO), carbohydrates (CHO), and lipids (LIP)] was analyzed using WebDiet® software (Rio de Janeiro, Brazil). Creatine intake was estimated from food records, considering its reported content and bioavailability in different food sources [22−24].

#### 2.3.5 **Statistical analysis**

Statistical analysis was performed on SAS 9.2® software (Institute Inc., Cary, NC). Data was analyzed using an intention-to-treat approach. A linear mixed model with repeated measures was performed using a restricted maximum likelihood algorithm under the missing at random assumption. Kenward–Roger degrees-of-freedom adjustment was used to adjust for data imbalance resulting from missing data. Absence of extreme observations (outliers) was guaranteed through standard visual inspection. Data normality and homoscedasticity was visually checked with histogram of the studentized residuals and residual plots; variables with skewed distributions were log-transformed prior to analysis to meet model assumptions. “Time” (T0, T1 and T2) was included as a fixed factor and “athletes” as a random factor with assumed normal distribution. A directed acyclic graph was used to visualize the causal relationships between the intervention, the outcome, and potential confounders (e.g. age, body mass index, menstrual status, contraceptive use, category, player position, and baseline values), which were included as covariates in the statistical model for adjustment (Supplementary Figure S1). Repeated covariance structures were specified as compound symmetry. Significance level was set at *p* ≤ 0.05. Descriptive data are presented as mean ± SD, unless stated otherwise. For longitudinal data, estimated means and 95% confidence intervals (95CI) from linear mixed models are reported. Whenever a significant *F* value was observed, pairwise comparisons were performed with Bonferroni’s *post-hoc* adjustment to control for multiple testing.

## 3 Results

Characteristics of participants are presented in Table 1. Adherence to supplementation was 100%. All participants were eumenorrheic, except for five athletes who reported being using oral contraceptives. Three, twelve, and four athletes reported being menstruated at the time of sample collection at T0, T1, and T2, respectively. Missing data was primarily due to scheduling conflicts during the regular season (e.g. competition calendar or unavailability for biochemical blood assessments), such as national team call-ups at T1 (*n* = 1) and T2 (*n* = 1), injuries (*n* = 1), or transfers to other clubs after baseline assessments (*n* = 3).

**Table 1.** Participants characteristics at baseline.

	Total (*n* = 71)	U17 (*n* = 13)	U20 (*n* = 25)	PRO (*n* = 33)
Age (years)	22 ± 6	16 ± 0	18 ± 1	27 ± 5
Sex (% females)	100	100	100	100
Body weight (kg)	60.81 ± 6.67	59.43 ± 6.23	59.36 ± 6.39	62.45 ± 6.66
Height (cm)	1.65 ± 0.06	1.62 ± 0.05	1.64 ± 0.06	1.67 ± 0.06
BMI (kg/m^2^)	22.24 ± 1.96	22.49 ± 2.23	22.24 ± 1.59	22.32 ± 2.07
*Position*				
Goalkeeper, *n* (%)	10 (14)	1 (8)	4 (16)	5 (15)
Defender, *n* (%)	25 (35)	5 (38)	10 (4)	10 (31)
Midfielder, *n* (%)	17 (24)	4 (31)	4 (16)	9 (27)
Forward, *n* (%)	19 (27)	3 (23)	7 (28)	9 (27)

Data are presented as mean ± SD or as the number of individuals (%). BMI: body mass index; U17: under-17 category; U20: under-20 category; PRO: professional athletes.

**Figure 1.**
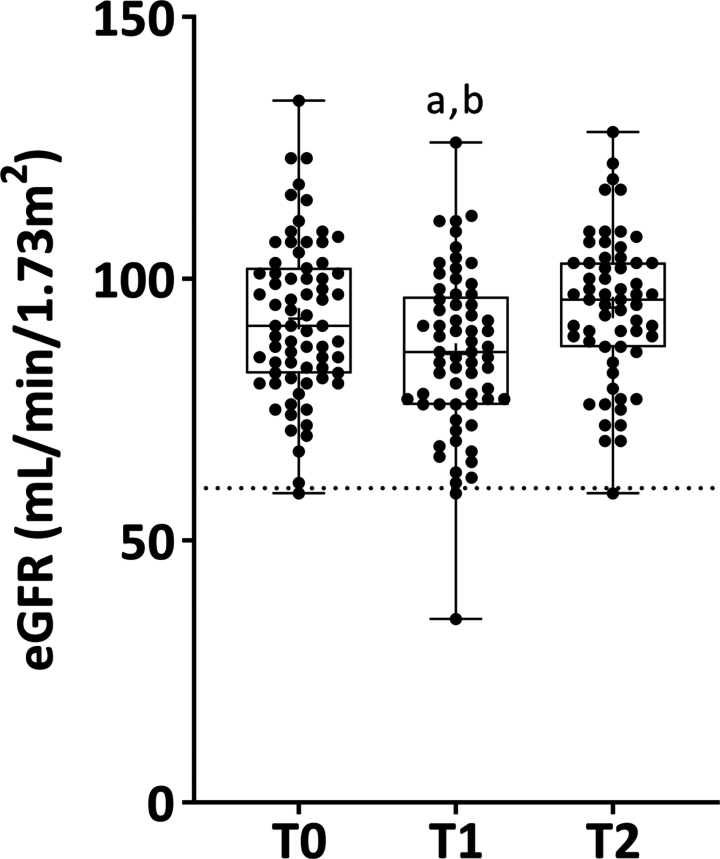
Results from linear mixed-model with repeated measures analysis adjusted for age, body mass index, menstrual status, contraceptive use, category, player position, and baseline values for the estimated glomerular filtration rate (eGFR) in 71 female football players. Data are presentend as median (lines), interquartile range (boxes), and individual data (black dotts) from minimum-to-maximum values (whiskers). Dotted line indicate the lower limit of reference. T0 (baseline): 90.34 mL/min/1.73m^2^ (95CI: 86.83 to 93.84); T1 (after 16 weeks): 83.57 mL/min/1.73m^2^ (95CI: 80.22 to 86.93); T2 (after 32 weeks): 91.58 mL/min/1.73m^2^ (95CI: 87.81 to 94.94). “a” indicate significant differences from T0 (*p* < 0.0001); “b” indicate significant differences from T1 (*p* < 0.0001).

Figure 1 shows eGFR response to creatine supplementation over time. eGFR slightly decreased at mid-season (T0 × T1: *p* < 0.0001; T1 × T2: *p* < 0.0001), and returned to basal levels at end-season (T0 × T2: *p* = 1.000). Results for other blood and urinary safety biomarkers are presented in Table 2. A main effect of time was found for seven out of 17 analytes (all *p* ≤ 0.001; Table 2). Creatinine and MCV increased at mid-season (T0 × T1: *p* < 0.0001 for both; T1 × T2: *p* < 0.0001 for both) and returned to basal levels at end-season (T0 × T2: *p* > 0.05 for both). An opposite pattern was observed for MCHC (T0 × T1: *p* = 0.0014; T1 × T2: *p* = 0.0007; T1 × T2: *p* = 1.000). RDW decreased from baseline (T0 × T1: *p* = 0.008; T0 × T2: *p* < 0.0001) and remained stable from mid- to end-season (T1 × T2: *p* = 0.617). K^+^ increased at mid-season (T0 × T1: *p* < 0.0001) and diminished at end-season (T1 × T2: *p* = 0.002) but did not return to basal levels (T0 × T2: *p* = 0.006). Albuminuria decreased over time (T0 × T1: *p* = 0.016; T0 × T2: *p* < 0.001; T1 × T2: *p* < 0.001). CPK had a significant increase at mid-season (T0 × T1: *p* = 0.04) followed by a significant reduction at end-season (T1 × T2: *p* < 0.0001); no difference was observed between baseline and end-season (T0 × T2: *p* = 0.13). No other statistical differences were observed. Comparable findings were observed when percentage change across time points were assessed (Supplementary Table 1).

**Table 2.** Effects of creatine supplementation on biochemical markers of female football players.

Biochemical markers	Reference (Range)	T0	T1	T2	Main effect of time	
					F-value	*p*-value
Sample size, n		71	67	66		
Erythrocytes, ×10^6^ µL	3.80–4.80	4.57 (4.48; 4.67)	4.56 (4.47; 4.65)	4.57 (4.47; 4.66)	0.12	0.891
Hematocrit, %	36.0–46.0	40.39 (39.64; 41.13)	40.71 (39.99; 41.42)	40.29 (39.52; 41.05)	1.15	0.320
Hemoglobin concentration, g/dL	12.0–15.0	13.49 (13.25; 13.74)	13.48 (13.25; 13.71)	13.50 (13.25; 13.75)	0.02	0.976
MCV, fL	83.0–101.0	88.58 (87.93; 89.23)	89.55 (88.92; 90.18)*	88.59 (87.92; 89.25)#	14.15	<.0001
MCH, pg	27.0–32.0	29.81 (29.54; 30.07)	29.75 (29.50; 30.00)	29.82 (29.55; 30.09)	0.32	0.727
MCHC, g/dL	31.0–35.0	33.58 (33.31; 33.84)	33.18 (32.93; 33.43)*	33.60 (33.33; 33.87)#	8.71	0.0003
RDW, %	11.6–14.0	12.98 (12.80; 13.15)	12.81 (12.64; 12.98)*	12.74 (12.56; 12.92)*	10.09	<.0001
White blood cell count, ×10^9^/L	4.50–13.00	6.38 (5.82; 6.95)	6.76 (6.22; 7.30)	6.82 (6.25; 7.40)	2.30	0.104
Platelet count, ×10^9^/L	150–450	273.8 (260.9; 286.7)	265.5 (253.2; 277.8)	271.3 (258.1; 284.6)	1.47	0.234
CPK, U/L^†^	34–145	124.4 (101.8; 151.9)	152.4 (126.0; 184.3)*	105.5 (85.9;129.5)#	9.76	0.0001
Creatinine, mg/dL	0.50–1.10	0.90 (0.87; 0.93)	0.95 (0.93; 0.98)*	0.89 (0.86; 0.92)#	21.51	<.0001
Albuminuria, mg/L^†^	≤20.00	5.16 (3.59; 7.41)	3.58 (2.53; 5.05)*	2.10 (1.46; 3.03)*,#	24.27	<.0001
Urea, mg/dL	19.3–49.2	30.95 (28.70; 33.20)	30.00 (27.85; 32.15)	29.65 (27.34; 31.95)	1.15	0.318
Na^+^, mmol/L	136–145	138.1 (137.5; 138.6)	138.4 (137.9; 138.9)	137.9 (137.3; 138.5)	1.39	0.254
K^+^, mmol/L	3.5–5.5	4.37 (4.25; 4.50)	4.80 (4.68; 4.92)*	4.57 (4.43; 4.72)*,#	37.75	<.0001
ALT, U/L	10–49	15.65 (12.71; 18.58)	17.30 (14.47; 20.13)	17.19 (14.19; 20.19)	1.60	0.206
AST, U/L	13–35	20.69 (18.51; 22.86)	21.80 (19.73; 23.87)	21.38 (19.16; 23.60)	0.87	0.423
Ferritin, ng/mL	10.0–291.0	65.62 (53.55; 77.69)	62.32 (48.18; 76.45)	65.96 (53.88; 78.03)	0.19	0.828

Results from linear mixed-model with repeated measures analysis adjusted for age, body mass index, menstrual status, contraceptive use, category, player position, and baseline values. Data are presented as an estimated mean (95% confidence interval). T0: baseline; T1: after 16 weeks; T2: after 32 weeks; MCV: mean corpuscular volume; MCH: mean corpuscular hemoglobin; MCHC: mean cell hemoglobin concentration; RDW: red blood cell distribution width; CPK: creatine phosphokinase; ALT: alanine aminotransferase; AST: aspartate aminotransferase. ^†^indicate log-transformed variables prior to analysis due to skewed distributions (results are reported as back-transformed values, i.e. geometric least-squared means and 95% confidence intervals); *indicate significant differences from T0 (*p* ≤ 0.05); ^#^indicate significant differences from T1 (*p* ≤ 0.05).

Table 3 shows players nutritional intake throughout the season. A main effect of time was found for all dietary variable (all *p* ≤ 0.001; Table 2). Compared to baseline, TEI showed a reduction only by the end of the season (T0 × T2: *p* < 0.001). Relative CHO intake increased at mid-season (T0 × T1: *p* = 0.002) but significantly reduced at end-season (T0 × T2: *p* < 0.0001; T1 × T2: *p* < 0.0001). Relative PTN intake decreased from baseline (T0 × T1: *p* < 0.0001; T0 × T2: *p* < 0.0001) and remained stable from mid- to end-season (T1 × T2: *p* = 1.000). Relative LIP intake fluctuated at mid-season (T0 × T1: *p* = 0.0001; T1 × T2: *p* < 0.0001), but no difference was found between baseline and end-season (T0 × T2: *p* = 0.317). Relative creatine intake increased from baseline as per protocol (T0 × T1: *p* < 0.0001; T0 × T2: *p* < 0.0001) and remained stable throughout the season (T1 × T2: *p* = 1.000).

**Table 3.** Dietary parameters during the intervention period.

				Main effect of time	
	T0	T1	T2	F-value	*p*-value
Sample size, n	34	34	34		
TEI (kcal/day)	1751.1 (1598.3; 1903.8)	1619.9 (1474.4; 1765.4)	1486.8 (1335.8; 1637.8)*	7.78	0.001
CHO intake (g/kg/day)	3.80 (3.34; 4.25)	4.30 (3.86; 4.74)*	2.78 (2.33; 3.22)*,#	31.14	<.0001
PTN intake (g/kg/day)	1.90 (1.76; 2.05)	1.20 (1.06; 1.33)*	1.23 (1.09; 1.37)*	54.30	<.0001
LIP intake (g/kg/day)	0.87 (0.75; 0.99)	0.65 (0.53; 0.76)*	0.96 (0.84; 1.08)#	18.73	<.0001
Creatine intake (g/kg/day)	0.02 (0.01; 0.02)	0.10 (0.10; 0.10)*	0.10 (0.09; 0.10)*	1235.79	<.0001

Results from linear mixed-model with repeated measures analysis adjusted for age, body mass index, menstrual status, contraceptive use, category, player position, and baseline values. Dietary intake could only be assessed for the U17 and U20 athletes. Data are presented as an estimated mean (95% confidence interval). T0: baseline; T1: after 16 weeks; T2: after 32 weeks; TEI: total energy intake; CHO intake: carbohydrate intake; PTN intake: protein intake; LIP intake: lipid intake. *indicate significant differences from T0 (*p* ≤ 0.05); ^#^indicate significant differences from T1 (*p* ≤ 0.05).

## 4 Discussion

This trial investigated the effects of long-term, in-season creatine supplementation on safety markers in female football players. Over the course of the 32-week season, seven out of 18 biochemical markers showed minor changes within clinical reference values, except for CPK. Overall, our findings indicate that creatine supplementation was not associated with clinically meaningful changes.

Creatine is one of the most recommended and well-established dietary supplements for enhancing sports performance [3,4], including football [5]. Nonetheless, anecdotal concerns persist regarding its potential adverse effects, particularly related to kidney function, liver health, and dehydration. The potential impact of creatine supplementation on kidney health has been extensively debated over the past 30 years (for a comprehensive review, see Longobardi et al. [17]). Indeed, creatine supplementation can affect creatinine metabolism, as creatinine is generated spontaneously and irreversibly from creatine through a non-enzymatic process. As a result, athletes who use creatine supplements may have higher serum creatinine levels without necessarily indicating an impaired renal function [25]. In our study, although eGFR slightly decreased at T1, fewer than 3% of participants exhibited abnormal values across all timepoints. Importantly, eGFR values returned to baseline values at T2. Because eGFR is derived from serum creatinine, it is unsurprising that a comparable pattern was observed for these biomarkers. However, it is noteworthy that even the 95CI upper limit remained well below the upper limit of reference values for serum creatinine (i.e. <1.10 mg/dL) at the same timepoint, supporting the safety of supplementation. This assumption is corroborated by the decrease of albuminuria and maintenance of urea levels over time, both within the reference ranges throughout the study.

Dietary creatine is also associated with liver metabolism, as its intake inhibits the activity of L-arginine:glycine amidinotransferase (AGAT) in the liver [25]. This reduces the production of guanidinoacetate and, consequently, the endogenous synthesis of creatine via guanidinoacetate methyltransferase (GAMT) [25]. Some studies in rodent models have shown that animals supplemented with creatine developed chronic hepatitis [26,27] and exhibited elevated levels of AST and ALT [28]. In contrast, the safety of creatine supplementation in humans with respect to liver function is further supported by several findings, as previously reviewed [29]. In the present study, no changes were observed in AST or ALT levels, extending this body of literature to female football players.

Due to its osmotic properties, creatine could influence hydration status by promoting water uptake into muscle fibers through a sodium-dependent creatine transporter [25]. This intracellular water retention could, in turn, reduce extracellular fluid availability, potentially leading to dehydration and electrolyte imbalances. Contrary to this, however, aside from minor and clinically insignificant changes observed in MCHC and K⁺ levels at T1, no other blood safety markers indicated any disruption in hydration status. These findings align with previous studies that found no changes in plasma volume [30,31], hematocrit [32], or electrolytes levels (e.g. Na^+^ and K^+^) [30] after creatine supplementation. While changes in MCV and RDW might suggest alterations in red blood cell characteristics, both mean values and confidence intervals remained well within reference. Although changes in MCV, MCHC, and RDW could also be attributed to alterations in iron status resulting from menstrual blood loss [33,34], this explanation seems unlikely since ferritin levels remained stable throughout the study and menstrual status was included as a covariate in the statistical model. Thus, these observations are likely due to random variation or normal physiological load-related fluctuations during the season [35].

CPK is widely used in clinical pathology as a marker of muscle enzyme efflux due to muscle fiber rupture, and in sports science as an indicator of muscular stress [36]. Although CPK levels showed a statistically significant increase at T1, it is noteworthy that several of the athletes were already above the upper limit of reference values prior supplementation at T0. Additionally, by the end of the season, most athletes had CPK levels within the reference range, despite the continued creatine supplementation. Thus, it is unlikely that creatine supplementation was the primary cause underlying CPK fluctuations throughout the season. In fact, a systematic review by Saidi et al. [37] reported that long-term soccer training led to moderate to very large increases in muscle damage markers such as CPK (effect size = 0.94–6.80). Therefore, various contextual factors, including pre- and in-season training load, periodization, session duration, and the competition schedule could have caused CPK levels to change over time [37].

Due to scheduling inconsistencies during the regular season, dietary intake could only be assessed in a subsample comprising U17 and U20 athletes. Our results showed considerable variability in dietary parameters across the season, which may also have influenced some of the biochemical markers. Notably, the relative intake of key macronutrients, such as CHO and PTN, was well below the recommended (and prescribed) for football players (e.g. CHO: 4–8 g/kg/day; PRO: 1.6–2.2 g/kg/day) [18]. Since this shortfall may have practical consequences for player performance, especially during the end-season when the most important matches occur, these findings emphasize the need for close monitoring of dietary nutrient intake in this population. Future studies should further investigate this issue and determine whether the observed variability truly exists or simply reflects difficulties in accurately completing food records, as reported by some of the athletes.

The main strengths of this study are its longitudinal design, spanning an entire football season, and the use of a well-defined sample of female football players and a broad assessment of safety markers. These factors strengthen the study’s internal validity and enhance the relevance of its findings to real-world athletic contexts. However, studying is not without limitations. Due to the challenges of implementing such interventions with high-performance athletes and the widespread use of creatine supplements among football players, a randomized placebo-controlled trial was not feasible. Since the CKD-EPI equation relies on serum creatinine, which is expected to rise after creatine intake, changes in eGFR may not accurately reflect actual renal function [17]. Additionally, due to limited data availability, we were unable to adjust our statistical model for other potential confounding factors (e.g. habitual diet, training load and periodization, psychological state, and sleep quality) that could have influenced some outcomes.

## 5 Conclusion

Although minor fluctuations in blood biochemical safety markers were observed throughout the season, all biomarkers remained within clinical reference values during long-term creatine supplementation. These results suggest that creatine supplementation is safe and well tolerated in female football players over the course of an entire competitive season. Our findings add to a large body of evidence supporting the safety of creatine across a broad range of individuals, from clinical populations to athletes. Future research should employ randomized, placebo-controlled designs to determine whether the observed differences are attributable to creatine supplementation or merely reflect seasonal effects.

## Supplementary Material

Supplementary Material Table S1

Supplementary Material Figure S1

## Data Availability

The data that support the findings of this study are available from the corresponding author upon reasonable request.
