# Correction

**DOI:** 10.1080/15502783.2025.2607167

**Published:** 2025-12-27

**Article title:** Safety of long-term creatine supplementation in women's football players: a real-world in-season study

**Authors:** Garcia, M. P., Longobardi, I., Saito, T., Miranda, M. S., Roschel, H., & Gualano, B.

**Journal:** *Journal of the International Society of Sports Nutrition*

**Bibliometrics:** Volume 22, Number S1**,** pages 1–9

**DOI:** http://dx.doi.org/10.1080/15502783.2025.2591782

This article was first published with the following errors:

1. Incorrect alignment of values in Table 2.
2. Incorrect Figure 1 legend description. “b” represents significant differences from T2 and not T1 as originally stated.

The article has been republished with the errors corrected.

