# Supplementary material for: Safety of long-term creatine supplementation in women's football players: a real-world in-season study
Source: J Int Soc Sports Nutr. 2025 Dec 2;22(Suppl 1):2591782. doi: 10.1080/15502783.2025.2591782 (PMC12673977; doi:10.1080/15502783.2025.2591782)
Supplement: Supplementary Material — Table S1 [file RSSN_A_2591782_SM8784.docx]

| **Supplementary Table 1.** Percentage change in biochemical markers across time points after creatine supplementation in female football players. | | | |
| --- | --- | --- | --- |
| **Laboratory Markers** | **T0×T1** | **T1×T2** | **T0×T2** |
|  |  |  |  |
| Sample size, n | 71 | 67 | 66 |
| Erythrocytes | −0.33 (−3.18; 2.52) | 0.16 (−2.73; 3.06) | −0.17 (−3.12; 2.78) |
| Hematocrit | 0.78 (−1.75; 3.31) | −1.03 (−3.59; 1.52) | −0.26 (−2.87; 2.36) |
| Hemoglobin concentration | −0.12 (−2.62; 2.38) | 0.13 (−2.40; 2.67) | 0.01 (−2.56; 2.59) |
| MCV | 1.09 (0.08; 2.10)^*^ | −1.08 (−2.09; −0.07)^*^ | 0.00 (−1.03; 1.04) |
| MCH | −0.19 (−1.42; 1.03) | 0.23 (−1.01; 1.48) | 0.04 (−1.22; 1.30) |
| MCHC | −1.18 (−2.26; 0.10) | 1.27 (0.16; 2.37)^*^ | 0.07 (−1.05; 1.19) |
| RDW | −1.30 (−3.14; 0.55) | −0.54 (−2.45; 1.36) | −1.83 (−3.74; 0.07) |
| White blood cell count | 5.92 (−6.22; 18.06) | 0.92 (−10.66; 12.50) | 6.90 (−5.66; 19.45) |
| Platelet count | −3.03 (−9.49; 3.43) | 2.19 (−4.54; 8.93) | −0.90 (−7.60; 5.80) |
| CPK | 4.21 (−1.51; 9.94) | −7.32 (−12.90; −1.75) ^*^ | −3.42 (−9.38; 2.53) |
| eGFR | −7.49 (−12.81; −2.18)^*^ | 9.34 (3.53; 15.15)^*^ | 1.15 (−4.34; 6.63) |
| Creatinine | 5.83 (1.72; 9.93)^*^ | −6.65 (−10.57; 2.73) | −1.21 (−5.45; 3.03) |
| Albuminuria | −24.6 (−61.2; 14.0) | −49.6 (−98.4; −1.2)^*^ | −62.0 (−99.6; −24.4)^*^ |
| Urea | −3.09 (−13.05; 6.85) | −1.16 (−11.56; 9.24) | −4.21 (−14.53; 6.14) |
| Sodium | 0.22 (−0.30; 0.74) | −0.30 (−0.87; 0.26) | −0.09 (−0.67; 0.49) |
| Potassium | 9.85 (5.89; 13.82)^*^ | −4.76 (−8.67; −0.86)^*^ | 0.20 (0.25; 8.99)^*^ |
| ALT | 10.57 (−15.3; 36.4) | −0.61 (−24.2; 22.9) | 9.90 (−16.6; 36.5) |
| AST | 5.37 (−8.99; 19.74) | −1.93 (−15.73; 11.87) | 3.34 (−11.53; 18.22) |
| Ferritin | −5.04 (−33.65; 23.60) | 5.84 (−24.31; 35.99) | 0.51 (−25.75; 26.74) |
| Data are presented as percentage change (%) and 95% confidence interval. T0: baseline; T1: after 16 weeks; T2: after 32 weeks; MCV: mean corpuscular volume; MCH: mean corpuscular hemoglobin; MCHC: mean cell hemoglobin concentration; RDW: red blood cell distribution width; CPK: creatine phosphokinase; ALT: alanine aminotransferase; AST: aspartate aminotransferase. * indicate significant differences (*p* ≤ 0.05). | | | |
