# Supplementary figures and images for: Safety of long-term creatine supplementation in women's football players: a real-world in-season study
Source: J Int Soc Sports Nutr. 2025 Dec 2;22(Suppl 1):2591782. doi: 10.1080/15502783.2025.2591782 (PMC12673977; doi:10.1080/15502783.2025.2591782)

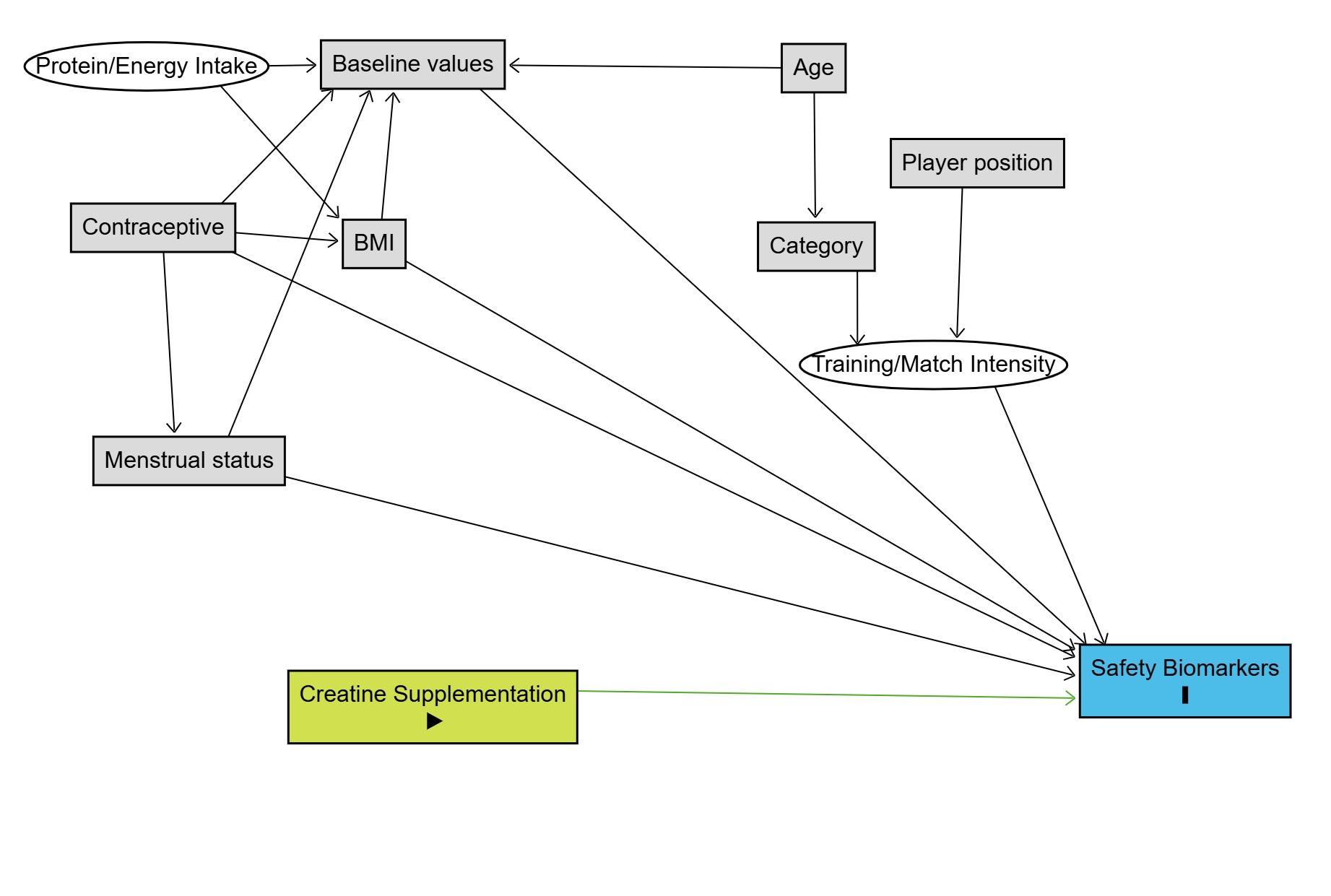

Supplement: Supplementary Material — Figure S1 [file RSSN_A_2591782_SM8785.jpeg]
